# The German System

**Published:** 1911-07-01

**Authors:** 


### THE GERMAN SYSTEM.

### II. THE PRESENT POSITION OF HOSPITALS IN GERMANY.

(By Our Special Commissioner.)

The German hospital system is not unique, nor does it stand apart, as some of us appear to imagine, from the rest of hospital systems by reason of its originality. It is merely the carefully worked out systematisation of a co-operation between State and municipality on the one hand, and between the communal authorities and voluntary bodies on the other, to obtain the best results for the sick within the German Empire. We say the sick, and not, as is usual when referring to hospitals in this country and America, the sick poor. The German idea of a hospital is an institution where the best treatment is afforded to the citizen who is unwilling or unable to make his own terms with a private doctor at a cost which is well within the means of the middle-classes and those who have been thrifty enough to provide for their days of sickness and disease. In the proper sense of the term there are no hospital poor in Germany; no patient is treated free of charge in a ward belonging to a public hospital: if he cannot pay for himself, his club or insuring society must pay, or if that cannot afford to do so, or if he has not joined some such organisation, the communal authorities must pay his hospital bill. In theory the last-mentioned provision operates exactly in the same way as does the pauper provision in this country; in practice it works quite differently. It is easy enough to see the immense advantage a hospital gains when it no longer bears the stigma of a pauper infirmary. Equally important, perhaps, is the moral effect of such a régime of paying patients upon those who are admitted to the wards. In the lower classes of wards—which are nevertheless paying wards in the strict sense of the term—the patients cannot differentiate between themselves; they cannot point out to Number so-and-so as a pauper patient who is being paid for by the ratepayers. Only the officials know the position and social standing of the patients, and in the eyes of their fellow-patients they are all equal and all enjoy the same privileges and the same comforts.

That being the case, it is evident that it would be unfair, in any broad purview of the subject, to institute a strict comparison between hospitals in this country and those in Germany. The difference of standing between them is reflected in other ways besides that of prestige. In general the German institution is a more sedate and steady place; it does not hurry itself unduly; the average length of stay of its patients is longer than in English hospitals of the same standing and size; its methods are in some ways uneconomical, not to say wasteful, and it provides for the immediate present and does not pay much attention to the future. The day-room in a German hospital is relatively large; the convalescent home, if it exists in connection with the institution, usually ridiculously small for the number of beds of the parent hospital.

But undoubtedly the main distinction between the German and the English hospitals is to be found in the relative position which they occupy in the public estimation. Here we are constantly and continually endeavouring to explain to the public the necessity of hospitals; in Germany it is an axiom, admitted as readily by the public as the corresponding axiom that isolation is necessary in cases of infectious disease, that hospitals are imperatively required, that they fulfil a useful object, and that they are worth supporting in every way. Hospital appeals in Germany are few and far between, and are confined to the class of private voluntary institutions which is rapidly undergoing a change, being in process of transformation into municipal institutions.

Hospitals in the German Empire may be divided, roughly, into four large classes: (1) Military hospitals and institutions officered, managed, and wholly supported by the State. With the exception of military lazarettes and a few special institutions, such as the leper colony at Memel, such hospitals are under the provincial State authorities, who are wholly responsible for their upkeep. Military and naval hospitals are supported either by the War Office of the Empire or by the provincial war-chests. (2) Public hospitals or institutions which are subsidised by the municipal or provincial State authorities, which are controlled by boards of management appointed by these authorities, and which receive their support almost entirely from the municipal treasuries. (3) Private hospitals belonging to charitable corporations, religious bodies, trusts, associations, or societies, and supported wholly or in part by voluntary effort, though they may be and often are subsidised by the municipality. They are managed by elective bodies chosen by the subscribers or others interested in the institution, and are for the most part religious institutions or endowed hospitals or Stiftungen. (4) Private clinics, belonging to a private individual or body of individuals, to a company or association, and run as business undertakings, though in some cases the amount of profit is so small and the undertaking so dependent upon voluntary support that the hospital may be claimed as an instance of charitable effort.

### The German Hospitals.

As examples of Class 1 may be cited the large military hospitals at Coblenz and Strassburg, which are analogous to our own at Netley and Aldershot. The second class is by far the most numerous, and in it are included the finest and best examples of hospital development in Germany, such as the splendid new city hospitals at Berlin (Moabit, Virchow, and Urban), at Hamburg (Eppendorf and St. George), at Stettin, Kiel, Breslau, Munich, Erlangen, and Gottingen. In the third class are comprised those hospitals which are the nearest analogies to our own voluntary institutions; they are usually old, but in many cases of strikingly interesting construction, admirably managed, and enjoying, as in the case of the Jewish Hospital at Berlin, a reputation for excellence of work which is second to none in the Empire. The fourth class needs merely a note of comment; in it are included all nursing and maternity homes. The reader should bear this in mind, since in German hospital statistics the institutions of the third and fourth classes are lumped together under the heading of "private hospitals of more than eleven beds."

Under the laws of the various States it is easy enough to establish a private voluntary hospital, on, say, the English model, but interesting provision is made for its continued existence. The Stifter, or endower, who gives the site and pays for the building and equipment, must obtain the sanction of the municipality within whose boundaries it is proposed to erect the institution. The plans and estimates must be submitted to the authorities, and a definite concession must be obtained before building operations can be commenced. Such a concession may be revoked by the central authority at any time if it has reason to believe that the institution is badly conducted. If communal necessities warrant it, the authorities may take over a semi-public hospital (but not a private clinic) and transform it into a rate-aided institution, or make themselves wholly responsible for its upkeep. Where the endowment is on behalf of and for the public, the institution must be properly made over to the authorities, and becomes a municipal hospital under the second class.

### Their Revenues.

A German hospital, then, may receive its revenue from many sources. The chief channels of income may be grouped as follows: (1) State support, in the shape of direct subsidies paid by the War Office or the Admiralty or some other department of State, either of the Imperial Government or of the Governments of the provincial States; such support is mainly confined to hospitals of the first class, though hospitals with polyclinics and medical schools attached receive subsidies from the Treasury. (2) Grants from municipalities. These are given not only to hospitals of the second class, but to those of the third as well, under certain conditions. Hospitals of the second class are almost wholly supported by such grants (see 3 and 4), which are made by the city councils on the recommendation of the city hospital office or sanitary committee which has charge of the institutions. For municipal hospitals the grant is calculated as follows: The ordinary net income of the institution is subtracted from the estimated expenditure for the year (to which is added depreciation and interest on building fund); the difference is the amount of the grant paid by the municipality; no special hospital rate is levied. In the case of voluntary hospitals the sanitary committee may, and sometimes does, recommend that a grant should be made in special cases, but the main help of the municipality is given in contributing towards the treatment of patients (see 3). (3) Patients' payments. Every patient who enters a hospital must pay; there is scarcely any exception to this rule, since even the purely religious and convent hospitals have adopted it; the exceptions are found in the case of maternity homes and orphan hospitals (belonging to the third class) in which provision is made under the will of the founders for the gratuitous treatment of certain cases. Generally the hospitals are divided into three classes of wards; in the first class (single-bedded wards) the patients pay from six marks upwards per day; in the second (double-bedded wards) the charge is from 3.50 marks per day; in the third or ordinary wards the charge is from 1 mark per day. Some hospitals have very much lower scales, but the scale is almost uniform in the municipal institutions. If the patient cannot pay, the municipality is debited with the cost of treatment, the charge in such cases being somewhat lower than the average daily rate charged for third-class patients. It is very difficult, in the absence of reliable statistics with regard to hospital income, to fix an average percentage of the amount contributed by patients, but it seems to us that in general it would be fair to take it that in the majority of State hospitals a fifth of the total expenditure is covered by such payments. (4) By proceeds from sale of refuse, oddments, etc. This item accounts for quite a large sum in the balance sheets of some of the big hospitals, like the Moabite or St. George. Nothing is wasted and all refuse is sold to contractors. (5) Bequests and donations: these are naturally a fluctuating amount and cannot be relied upon. (6) Voluntary contributions, subscriptions by individuals or private bodies for the upkeep of the institutions. These are mainly of benefit to the institutions in the third class. Some of the big firms have their own hospitals, which may be considered as private hospitals, although they receive outside contributions and can, therefore, hardly be classed under private clinics. The main source of voluntary contributions, however, is the Church. Many religious bodies have special hospitals, and get up special collections for them, while others devote a portion of their endowed funds towards their upkeep.

### Their Control by the State.

With regard to the State control of the hospitals, although such control is prescribed by law, in practice the State interferes very little indeed in the management of even the municipal hospitals. The district authorities have the right to inspect and report on all hospitals, but their powers are analogous to those of our own medical officers of health, sanitary and factory inspectors, who have in this country the right of entering a public hospital and inspecting certain parts of it. There is a central health authority for the whole Empire. This is the "Kaiserliche Gesundheitsamt," at 27 Luisenstrasse, at the head of which is a permanent director-president, who is a lawyer, and who is advised by a consultative board or council of thirty experts in medicine, hygiene, sanitation, etc. They are appointed by Imperial decree, and the director is empowered to ask further expert assistance in special cases. The Gesundheitsamt is for the most part a collecting station and an inquiry department; it has nothing to do with hospital work, which is purely an affair for the provincial authorities, but confines its attention mainly to statistical research and to reports, though it has, of course, a general supervision over the whole medical system within the Empire. It was established in 1876, and is under the direct control of the Chancellor; at present it is divided into four special departments (chemical, bacteriological, medical, and veterinary) and publishes interesting statistics relative to the health of the Empire.

### Provincial Control.

The authority in the different Bundesstaten varies. Nearly every province has a central State authority, usually the Minister for Education or for the Interior, who is entrusted with the general supervision of the State medical service, of medical education and qualification generally, and of the hospitals. The functions and duties of these authorities are regulated by special Acts of Parliament, for full details of which the reader is referred to the authoritative work on the subject (A. Guttstadt, Deutschlands Gesundheitswesen, Leipsig, 1899). As a general rule the Minister in charge delegates his functions so far as public health is concerned to his advisory committee, the head of which is usually a recognised sanitary or medical expert, or a lawyer assisted by a vice-president who is a qualified medical man. The authority regulates the duties of district surgeons, supervises examinations for medical men and midwives, prescribes the forms to be used in medico-legal cases, and deals with disciplinary cases. Besides this central authority there is usually in the same State a central city authority for each large municipal centre. This is the "Stadtischer Gesundheitsrath," which is the analogy of our own municipal health or hospital committee, and which has the control and supervision of the whole medical and sanitary system of the municipality. Under it are the city hospitals, whose staff, both medical and lay, are appointed or removed by it, and its rulings are subject only to the ratification of the general council, which is very rarely adverse. In Prussia and a few other States there are also provincial councils, similar in function, which control the large provincial institutions not directly under city councils; they have special powers granted by Act of Parliament. In Bavaria the Minister of the Interior is the central authority, and acts upon the advice and with the assistance of a specially appointed medical council. In Saxony the Minister of the Interior is also the authority, and is assisted by an advisory Landes-Medicinal-Kollegium; so also in Wurtemburg and Baden. The officials of the Kollegium have the right of inspecting all public hospitals, and to report on their sanitary condition. In Baden the Bezirksarzt is bound to visit every hospital, whether public or private, at least once every year and to report fully on its condition and management. He must also inspect every new institution, plans and a full description of which must be lodged with the Curatorium before it can be sanctioned for the admission of patients. Full official instructions are published with regard to the details of the hospital inspection, but no provision is made for the simultaneous inspection of institutions visited by the Bezirksarzt by architectural or hospital experts, with the results that the reports on hospitals are usually stereotyped, uninteresting, and by no means of any great value to the hospital worker. In Hessen the central authority delegates considerable powers of inspection to the Bezirksarzt, but in his instructions (Landesherrl. Verordnung of July 14, 1884) he is specifically warned that his inspection is merely to safeguard the public interest, that it should be confined to a scrutiny of the sanitary aspect of the institution, and that he is not concerned with the financial aspect. In Mecklenburg-Schwerin, the Minister of Justice is the central authority, and carries out his special duties with the help of an advisory medical council at Rostock. In Sachsen-Weimar a similar system obtains, while most of the other States follow the Prussian example. The free cities (Hamburg, Bremen, and Lubeck) have special city medical council or committees which possess extensive powers and are subject only to the City Councils; these committees practically act as the boards of management of the large hospitals belonging to these towns.

### Hospital Statistics.

By special resolution of the Bundesrat, passed in October 1875, complete statistical tables of all the charitable institutions within the Empire are kept. These records, unfortunately, do not give the financial status of the hospitals, and are available only for institutions possessing more than ten beds. All hospitals are classed under two headings : Private hospitals or institutions not directly controlled by and belonging to public bodies such as a municipality, and including private nursing homes; and public hospitals, which include all others, with the exception of military or naval lazarettes. Statistical tables are published from time to time in the official "Statistik für das Deutsche Reich," and every year in the "Statistisches Jahrbuch für das Deutsche Reich : Herausgegeben vom Kaiserlichen Statistischen Amte" (the latest edition of this which contains hospital figures is that of 1909). The following table gives the official figures for the years immediately before and after the introduction of the insurance laws. (The figures in brackets give the number of beds.)

### Table of German Hospitals.

| | 1877 | | 1888 | | 1901 | | 1904 | |
|---|---|---|---|---|---|---|---|---|
| Private Hospitals | 316 | (10,079) | 586 | (24,723) | 1,264 | (56,963) | 1.332 | (68.747 |
| Public Hospitals | 1,806 | (62.140) | 1,803 | (82,979) | 2,076 | (119,524) | 2,106 | (120,600) |

**The majority of private hospitals are, of course, private nursing homes, and not by any means voluntary hospitals depending on public support.** The increase in the number of private beds is due to the facilities for the erection of such homes which the law provides.

At present the number of beds in the Prussian hospitals works out at one for every 323 head of population, the figure for the whole Empire being 1 bed for every 322 head of population. Before the introduction of the insurance laws the figure was approximately one bed for every 800 persons. At Berlin the number of beds is still too small, being approximately one for every 500 (the figure for Paris is five beds for every 1,000 of population). In Hamburg there is one bed for every 180 inhabitants. In public institutions 63 per cent. of beds are in constant use; in private hospitals only 56 per cent. The increase in the private hospitals is well seen in the following table of general hospitals :—

| | 1893 | 1899 | 1900 | 1901 |
|---|---|---|---|---|
| Private Hospitals ... | 1,003 | 1,057 | 1,117 | 1,264 |
| Public Hospitals ... | 1,962 | 1,988 | 2,029 | 2,076 |
| Total | 2,965 | 3,045 | 3,146 | 3,340 |

In 1901 more than 41 per cent. of all the institutions were private, and 28 per cent. of all the beds were private. A general hospital is found in Germany for approximately every 17,000 of population; in Bavaria there is a hospital for every 12,000; in Hessen one only for every 22,000. No State was without a public hospital in 1901, but in that year there were no private hospitals in ten States. In four years private institutions increased 26 per cent., public hospitals only at the rate of 5 per cent. During the decade 1892-1901 public hospitals increased 9 per cent., while private hospitals increased at the rate of 79 per cent. The average stay per patient in the public hospitals is 28 days; in the private over 35 days; the majority of patients in private hospitals are female patients.

### Voluntary Aid and the Hospitals.

It is extremely difficult to get the figures for the private hospitals in Germany especially tabulated in such a way as to serve as a basis of comparison with those of the public institutions. The finances of the public hospitals, too, are not always collated, so that it is difficult, and in many cases impossible, to compare city hospitals with private institutions. No attempt, so far as we know, has been made as yet to tabulate the financial statistics of the German hospitals in order to show the differences between the revenues of the two classes. Guttstadt * has elaborated the statistics available, in order to show the differences of expenditure between the two classes; his tables are very interesting, but too long to be reprinted here.

The figures for the public hospitals show a gradually and steadily increasing total income derived from municipal subsidies and from patient's payments. The cost per bed per day has been worked out to the following averages by Grotjahn :—

For hospitals with 20 to 50 beds, 1.60 mark.
For hospitals with 50 to 150 beds, 1.53 to 1.55 mark.
For hospitals with 150 to 300 beds, 1.95 mark.
For hospitals with more than 300 beds, 3.39 marks.

The total income of the German hospitals, public and private, during the year 1901 is estimated (Guttstadt) at 123 million marks; the total expenditure at 120 million marks. Of this revenue three-fourths was supplied by the municipalities. The amount obtained from the insurance societies was as follows for the years 1906-07 :—

Expenditure of Krankenkassen on hospital treatment given in 1,000 marks :—

| | Sickness Insurance | Accident | Invalidity |
|---|---|---|---|
| 1905 ... ... ... ... | 34,170.5 | 1,264.1 | 349.7 |
| 1906 ... ... ... ... | 37,205.1 | 1,281.9 | 407.7 |
| 1907 ... ... ... ... | 41,246.5 | 1,312.0 | 444.0 |

Some nineteen insurance societies and five Krankenkassen have their own hospitals.

* A. Guttstadt. Die Betriebskosten der Offentlichen Heilanstalten und die Verpflegungssätze für Kranke. Zeitschrift für Sociale Medicin, 1906. See also the same author's book, "Die Universitätsstadte und die Universitätskliniken in Preussen," Berlin, 1903, and Helbig's interesting article, "Die Betriebsergebnisse der grossen Krankenanstalten im Jahre 1903," which appeared in the "Zeitschrift für Krankenanstalten, 1905. Heft 21"; and, further, Von Lindheim's book, "Saluti aegrorum, 1905," which gives detailed statistics for both German and Austrian clinics.

